# Clinical Significance of Metastasis or Micrometastasis to the Lymph Node Along the Superior Mesenteric Vein in Gastric Carcinoma: A Retrospective Analysis

**DOI:** 10.3389/fonc.2021.707249

**Published:** 2021-07-29

**Authors:** Xing Xu, Guoliang Zheng, Tao Zhang, Yan Zhao, Zhichao Zheng

**Affiliations:** ^1^Cancer Hospital of China Medical University, Liaoning Cancer Hospital & Institute, Shenyang, China; ^2^Department of Gastric Surgery, Cancer Hospital of China Medical University, Liaoning Cancer Hospital & Institute, Shenyang, China

**Keywords:** prognosis, gastric carcinoma (GC), micrometastases, metastasis to the lymph node, immunohistochemistry (IHC)

## Abstract

**Background:**

The validity of lymphadenectomy of the lymph node along the superior mesenteric vein (LN14v) in gastric cancer remains controversial. The study investigated the characteristics and prognosis of gastric cancer with metastasis or micrometastasis to LN14v.

**Methods:**

A retrospective study of 626 patients undergoing radical gastrectomy in our center from January 2003 to December 2015 was analyzed. In total, 303 patients had lymphadenectomy of LN14v, and lymph node micrometastasis was evaluated by immunohistochemical staining for cytokeratin nodes CK8/18. A logistic regression model was applied to confirm the predictive factors of micrometastasis. Survival analysis was performed to evaluate the effect of micrometastasis or metastasis on prognosis.

**Results:**

The metastatic rate of the LN14v lymph node was 15.8%, and the micrometastatic rate was 3.3%. Multivariate analysis showed site, Borrmann classification, postoperative lymph node metastasis (pN), and metastasis in LN6 and LN9 were predictive factors for LN14v micrometastasis or metastasis (*P* < 0.05). The 5-year survival rate in the positive group (LN14v micrometastasis or metastasis) was 12.4%. The prognosis of patients without LN14v lymph node micrometastasis was better than that of the positive group, whereas the difference between group of LN14v micrometastasis and LN14v metastasis was not obvious. In matched analysis, patients with stage III gastric cancer L/M area, pN2-3, and LN6(+) who underwent lymphadenectomy of LN14v had better survival than those without lymphadenectomy of LN14v.

**Conclusion:**

Lymph node micrometastasis may provide accurate prognostic information for patients with gastric cancer. Moreover, lymphadenectomy of LN14v might improve the survival of patients with stage III gastric cancer of L/M area, pN2-3, and LN6(+).

## Introduction

Gastric cancer (GC) is one the most common cancer-related cause of death throughout the world (1, 2). Radical gastrectomy is the optimal choice to cure locally advanced resectable GC. The 15-year results of a Dutch trial showed that D2 lymphadenectomy was associated with a lesser rate of recurrence and improved the overall survival than did D1 dissection, and gastrectomy plus D2 lymph node dissection has been increasingly considered as the standard surgical procedure for advanced resectable gastric cancer (3). However, over 50% of patients who had worse survival subsequently relapsed or died after radical surgery because of lymph nodes metastasis, distant metastasis, or locoregional recurrence (4). Among the metastatic routes in GC (direct infiltration or spread, lymph node metastasis, hematogenous metastasis, and implantation metastasis), lymph node metastasis remains the most common pathway. However, the extent of lymphadenectomy during the surgery that will maximize survival with few complications remains controversial.

According to the 6^th^ edition of International Union Against Cancer (UICC), lymph node metastasis (LNM) is classified into isolated tumor cells (ITC), micrometastasis (MI), and macrometastasis (MA), depending on the size of the metastatic deposit (5). Methods to investigate the presence of MI vary, from serial slices with hematoxylin and eosin (H&E) staining, immunohistochemical (IHC) staining, to real-time reverse transcription-polymerase chain reaction (RT-PCR) (6–9). Cytokeratin is one component of the cytoskeleton of epithelial cells that is not present in normal lymph nodes, and its corresponding antibody is widely used to detect minute deposits of tumor cells in lymph nodes by IHC staining. Controversies remain regarding the clinical features of MI and its prognostic significance for GC (10–13).

One particular study, the JCOG9501 trial, did not conclude that D2 lymphadenectomy plus para-aortic lymph node dissection improves survival in comparison to D2 lymphadenectomy alone (14, 15). Furthermore, the necessity for patients with GC to undergo LN14v dissection and how to identify the subgroup who received maximum benefit from LN14v dissection remain controversial. The current study was therefore designed to analyze the characteristics associated with LN14v metastasis or micrometastasis and the clinical features and prognostic significance of MI for GC. In addition, oncological outcomes were also analyzed.

## Methods and Materials

### Patients

From January 2003 to December 2015, 626 patients with GC (including esophagogastric junction carcinoma) in the Cancer Hospital of China Medical University, Liaoning Cancer Hospital, were prospectively enrolled in this study (**Supplementary Figure 1**). To analyze the characteristics associated with LN14v metastasis or micrometastasis, the inclusion criteria were as follows: (1) age range from 18 to 75 years old, (2) histopathological examination diagnosis of gastric carcinoma based on well-established criteria, (3) intraoperative exploration and postoperative tumor-node-metastasis (TNM) stage revealed no distant metastases (M0), (4) no conditions preventing resection were found, and (5) radical gastrectomy plus the LN14v lymph node dissection was performed (D2 or D2+). The study exclusion criteria were as follows: (1) R0 surgery after conversion therapy, (2) gastric remnant carcinoma, (3) LN14v lymph node biopsy, (4) presence of other malignancies, and (5) lack of complete follow-up data. A total of 303 patients receiving radical gastrectomy plus LN14v lymph node dissection were retrospectively identified for comparison between groups. Patients with clinical or pathologic TNM stage II-III or with positive lymph nodes received a perioperative chemotherapy regimen of SOX or XELOX, divided into two to four preoperative and several postoperative cycles. There were 255 patients without LN14v lymph node metastasis who underwent CK8/18 immunohistochemistry to detect minute deposits of tumor cells in the lymph nodes. Patients without micrometastases in the lymph nodes were classified as the negative group, and patients with micrometastases or metastases in the lymph nodes were classified as the positive group. This study was approved by the Ethics Committee of Liaoning Cancer Hospital.

### LN14v Lymph Node Dissection

The LN14v lymph nodes are classified as lymph nodes along the superior mesenteric vein. All radical gastrectomies plus the LN14v lymph node dissection were performed by three experienced surgeons. The dissection criteria for the LN14v lymph nodes were as follows: First, omentobursectomy and lymphadenectomy of LN6 was performed, and then LN14v lymph nodes were completely removed from the root of lymph nodes. In order to expose the superior mesenteric vein, Henle trunk, and middle colic vein, the soft tissue around the superior mesenteric vein was completely removed as well.

### Clinicopathological Features

Patients’ clinicopathological characteristics included tumor size, gastric location (upper [U], middle [M], lower [L] area), grade of differentiation (differentiated, undifferentiated), Borrmann type, histological type (adenocarcinoma, signet ring, or mucinous carcinoma), pathologic TNM stage (postoperative category), extent of resection (total, distal, or proximal), and the number of harvested lymph nodes.

### Immunohistochemical Staining

A total of 576 lymph nodes without LN14v metastasis from 255 patients were reexamined by one pathologist to confirm the absence of lymph node metastasis by H&E staining. Lymph nodes were stored in 261 paraffin-embedded blocks and three serial tissue sections 4 mm thick were cut from each block, and immunohistochemical staining (CK8/18) was performed to evaluate lymph node metastasis. The tumor tissues of GC were used as the positive control for staining in the same manner as the experimental group (**Supplementary Figure 2**).

### Assessing Staining Results

We evaluated the staining results according to the location, structure, morphology, and staining color. CK8/18 was mainly located in the cytoplasm at the marginal sinus of the lymph nodes (**Supplementary Figure 2**). Positive-staining cells were brown-yellow in color, while the negative cells were unstained. Furthermore, positive samples were reconfirmed by observing the structure and morphology of the cells. Only if the size of the cell nest was 0.2–2 mm was it defined as MI and the sample was classified as positive sample. Any serial sections with positive staining were categorized as the positive group. All of the slices were reviewed by two experienced pathologists who independently observed the CK-stained sections under the microscope (×100 and ×200). Any disagreements between the pathologists were resolved by consensus following a review of the samples.

### Statistical Analysis

Mean ± standard deviation was used to represent continuous values, and categorical data were expressed as percentages. Chi-square test or Fisher’s test was applied to evaluate the relationship between clinicopathological characteristics and LN14v metastasis or micrometastasis variance. Independent factors associated with LN14v metastasis or micrometastasis were analyzed by logistic regression. Variables with a *P* value of 0.05 in univariate analysis were selected for the multivariate analysis. To predict prognostic risk factors, we used the multivariable Cox proportional hazard model and univariate analysis. The Kaplan–Meier method and log-rank test were applied to distinguish differences in survival data between two groups. Data were processed by SPSS 23.0 software and GraphPad Prism 7.0. And *P* value <0.05 was considered to be statistically significant.

## Results

### Clinicopathological Features and Clinical Outcomes

A total of 303 patients underwent radical surgery plus LN14v dissection in the study, and the clinicopathological features are shown in **Table 1**. Most patients were male (66.0%), and their age ranged from 28 to 75 years old (mean: 55.9). A majority of tumors were located in the L area (67.7%), were poorly differentiated (77.2%), and were of the adenocarcinoma histological type (73.9%). A minority of patients underwent total or proximal gastrectomy (24.1%). Cases were classified into stage I (17.5%), II (20.1%), or III (62.4%) based on postsurgical pathology. The incidence of metastasis in LN14v lymph nodes was 15.8%, and the incidence of micrometastasis was 3.3%. The total metastatic rate in LN14v was 19.1%. The median number of overall harvested lymph nodes and harvested LN14v lymph nodes was 28.5 and 2.0, respectively. Patients were followed up every 3 months during the first 3 years, subsequently every 6 months for the following 2 years, and once a year after 5 years until the time of death or the study deadline, December 31, 2018. The follow-up time ranged from 3 to 178 m (median: 46 m). The 3- and 5-year overall survival rates of patients with radical gastrectomy plus LN14v dissection were 71.2 and 50.7%, respectively.

**Table 1:** Clinicopathological features of patients with gastric cancer undergoing radical gastrectomy plus 14v dissection.

Clinicopathological characteristics	Value	Percentage (%)
Age (y)	55.9±10.74	
Gender		
Male	200	66.0
Female	103	34.0
Tumor size (cm)	6.0±10.74	
Location		
U	14	4.6
M	84	27.7
L	205	67.7
Histological type		
Adenocarcinoma	224	73.9
Signet-ring or mucinous carcinoma	79	26.1
Grade of differentiation		
Well or moderate	69	22.8
Poor	234	77.2
Borrmann type		
I, II	178	58.7
III	108	35.6
IV	17	5.6
Postoperative T category (pT)		
T1-2	81	26.7
T3-4	222	73.3
Postoperative N category (pN)		
N0	89	29.4
N1	41	13.5
N2	63	20.8
N3	110	36.3
Extent of resection		
Total or proximal	73	24.1
Distal	230	75.9
Pathological stage (pTNM)		
I	53	17.5
II	61	20.1
III	189	62.4
The number of harvested lymph nodes	28.5±10.52	
The number of metastatic lymph nodes	6.3±7.51	
Without 14v micrometastasis	245	80.9
With 14v micrometastasis	10	3.3
With 14v metastasis	48	15.8
The number of harvested lymph nodes of 14v	2.0±1.439	

Location, U/M/L, the upper/middle/lower third of stomach.

### Clinicopathological Characteristics Associated With LN14v Metastasis or Micrometastasis

We sought to identify subgroups that were likely to have LN14v metastasis or micrometastasis. We found that metastasis or micrometastasis in LN14v was associated with tumor size (*P* = 0.001), location (*P* = 0.027), Borrmann type (*P* = 0.003), pT category (*P* < 0.001), pN category (*P* < 0.001), pTNM stage (*P* < 0.001), and the number of metastatic lymph nodes (*P* < 0.001) (**Table 2**). In regard of multivariate analysis, logistic regression analysis demonstrated that location (*P* = 0.004, RR: 0. 320, 95% CI, 0.146–0.700), Borrmann type (*P* = 0.010, RR: 1.519, 95% CI, 1.104–2.089), and pN category (*P <*0.001, RR: 3.709, 95% CI, 2.326–5.914) were significantly correlated with metastasis or micrometastasis in LN14v (**Table 3**).

**Table 2:** Univariate analysis of clinicopathological features associated with 14v metastatic status.

Variable	14v micrometastasis (−)	14v micrometastasis or metastasis (+)	*P* value
Age (y)			
<60	159	36	0.686
≧60	86	22	
Gender			
Male	162	38	0.930
Female	83	20	
Tumor size (cm)			
<5.0	127	16	0.001
≧5.0	118	42	
Location			
U	14	0	0.027
M	73	11	
L	158	47	
Histological type			
Adenocarcinoma	179	45	0.480
Signet-ring or mucinous carcinoma	66	13	
Grade of differentiation			
Well or moderate	57	12	0.674
Poor	118	46	
Borrmann type			
I, II	154	24	0.003
III	81	27	
IV	10	7	
Postoperative T category (pT)			
T1-2	77	4	0.000
T3-4	168	54	
Postoperative N category(pN)			
N0	89	0	0.000
N1	37	4	
N2	55	8	
N3	64	46	
Extent of resection			
Total or proximal	62	11	0.310
Distal	183	47	
Pathological stage(pTNM)			
I	52	1	0.000
II	59	2	
III	134	55	
The number of harvested lymph nodes	28.90±10.419	26.72±10.843	0.157
The number of metastatic lymph nodes	4.56±6.034	13.55±8.728	0.000
The number of harvested lymph nodes of 14v			
1	120	33	0.256
2	57	15	
≧3	68	10	

Location, U/M/L, the upper/middle/lower third of stomach.

**Table 3:** Multivariate analysis of clinicopathological features associated with 14v metastatic status.

Variables	*β*	RR(95% CI)	*P* value
Tumor size	0.331	1.392 (0.649–2.988)	0.396
Location	−0.139	0.320 (0.146–0.700)	0.004
Borrmann type	0.418	1.519 (1.104–2.089)	0.010
Postoperative T category (pT)	0.624	1.866 (0.565–6.160)	0.306
Postoperative N category (pN)	1.311	3.709 (2.326–5.914)	0.000
Pathological stage (pTNM)	−0.747	0.474 (0.097–2.313)	0.356

### Regional Lymph Nodes Associated With LN14v Metastasis or Micrometastasis

In order to investigate the LN14v lymphatic drainage pathway, the study included lymph nodes 1–4 (4sa, 4sb, and 4sd), 5–7, 8a, 9–11, and 12a for univariate and multivariable analysis. The analyses showed that the metastatic status of LN14v was significantly correlated with that of all regional nodes (all *P <*0.05, **Table 4**). Multivariable analysis results revealed the metastasis of LN6 and LN9 to be independent variables associated with LN14v metastasis or micrometastasis (*P* = 0.003, RR: 0.101, 95% CI, 0.022–0.496; *P* =0.013, RR: 0.093, 95% CI, 0.014–0.608) (**Table 5**). Of 146 patients with LN6 metastasis, 34.2% cases had metastasis or micrometastasis in LNLN14v. LN6 status had a low false-negative rate (10.7%) in predicting the absence of metastasis or micrometastasis in LN14v.

**Table 4:** Univariate analysis of regional lymph nodes associated with 14v metastatic status.

Lymphatic metastasis	14v micrometastasis (−)	14v micrometastasis or metastasis (+)	*P* value
No. 1			
(+)	23	17	0.000
(−)	179	30	
No. 2			
(+)	10	1	0.001
(−)	41	3	
No. 3			
(+)	90	39	0.000
(−)	142	11	
No. 4			
(+)	79	37	0.000
(−)	154	14	
No. 5			
(+)	46	29	0.000
(−)	103	9	
No. 6			
(+)	96	50	0.000
(−)	135	6	
No. 7			
(+)	63	29	0.000
(−)	167	20	
No. 8a			
(+)	42	40	0.000
(−)	189	11	
No. 9			
(+)	15	23	0.000
(−)	178	22	
No. 10			
(+)	1	2	0.005
(−)	10	0	
No. 11			
(+)	16	17	0.000
(−)	164	17	
No. 12a			
(+)	14	17	0.000
(−)	127	19	

No. 1, right paracardial lymph node; No. 2, left paracardial lymph node; No. 3, lymph node along the lesser curvature; No. 4 (4sa, 4sb, 4sd), lymph node along the short gastric vessels, the left gastroepiploic vessels, and the right gastroepiploic vessels; No. 5, the suprapyloric lymph node; No. 6, the infrapyloric lymph node; No. 7, lymph node along the left gastric artery; No. 8a, lymph node along the common hepatic artery; No. 9, lymph node around the celiac artery; No. 10, lymph node at the splenic hilum; No. 11 (11p and 11d), lymph node along the proximal splenic artery and distal splenic artery; No. 12a, lymph node in the hepatoduodenal ligament (along the hepatic artery); No. 14v, lymph node along the superior mesenteric vein.

**Table 5:** Multivariate analysis of regional lymph nodes associated with 14v metastatic status.

Lymphatic metastasis	*β*	RR(95%CI)	*P* value
No. 1	1.378	3.968 (0.340–46.257)	0.271
No. 3	−0.172	0.842 (0.054–13.085)	0.902
No. 4	1.464	4.325 (0.206–90.878)	0.346
No. 5	−0.189	0.150 (0.010–2.203)	0.167
No. 6	−2.294	0.101 (0.022–0.496)	0.003
No. 7	1.062	2.891 (0.118–71.054)	0.516
No. 8a	−1.395	0.248 (0.037–1.681)	0.153
No. 9	−2.38	0.093 (0.014–0.608)	0.013
No. 11	2.048	7.750 (0.203–295.209)	0.270
No. 12a	−0.345	0.708 (0.029–17.523)	0.833

No. 1, right paracardial lymph node; No. 3, lymph node along the lesser curvature; No. 4 (4sa, 4sb, 4sd), lymph node along the short gastric vessels, the left gastroepiploic vessels, and the right gastroepiploic vessels; No. 5, the suprapyloric lymph node; No. 6, the infrapyloric lymph node; No. 7, lymph node along the left gastric artery; No. 8a, lymph node along the common hepatic artery; No. 9, lymph node around the celiac artery; No. 11 (11p and 11d), lymph node along the proximal splenic artery and distal splenic artery; No. 12a, lymph node in the hepatoduodenal ligament (along the hepatic artery); No. 14v, lymph node along the superior mesenteric vein.

### Prognostic Value of Metastatic Status of LN14v in Gastric Cancer

**Figure 1:**
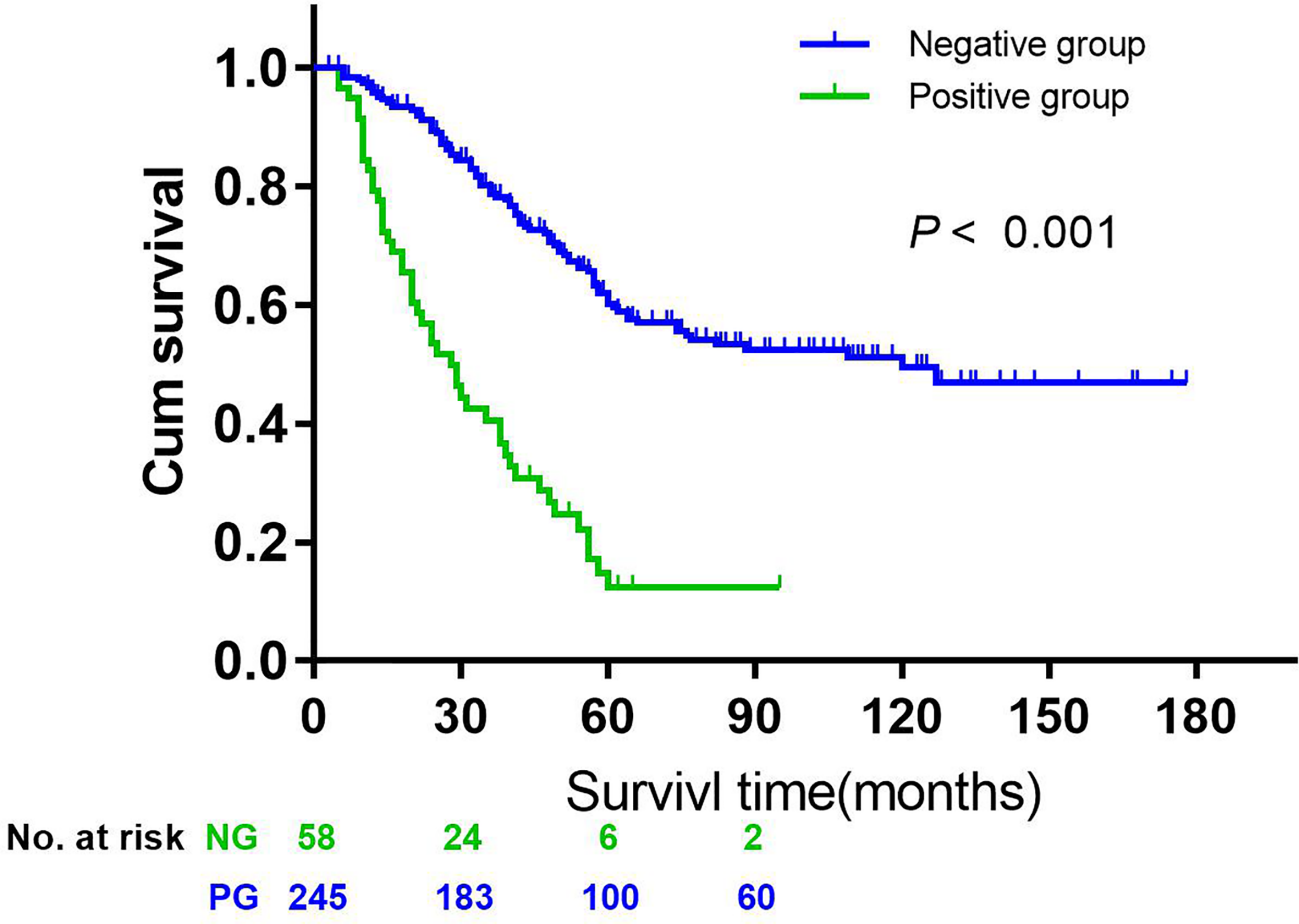
Kaplan-Meier curves were employed to compare the overall survival data between the negative group (without 14v micrometastasis and metastasis) and the positive group (with micrometastasis or metastasis) (*P* < 0.001, HR = 4.001, 95% CI = 2.789–5.739).

**Figure 2:**
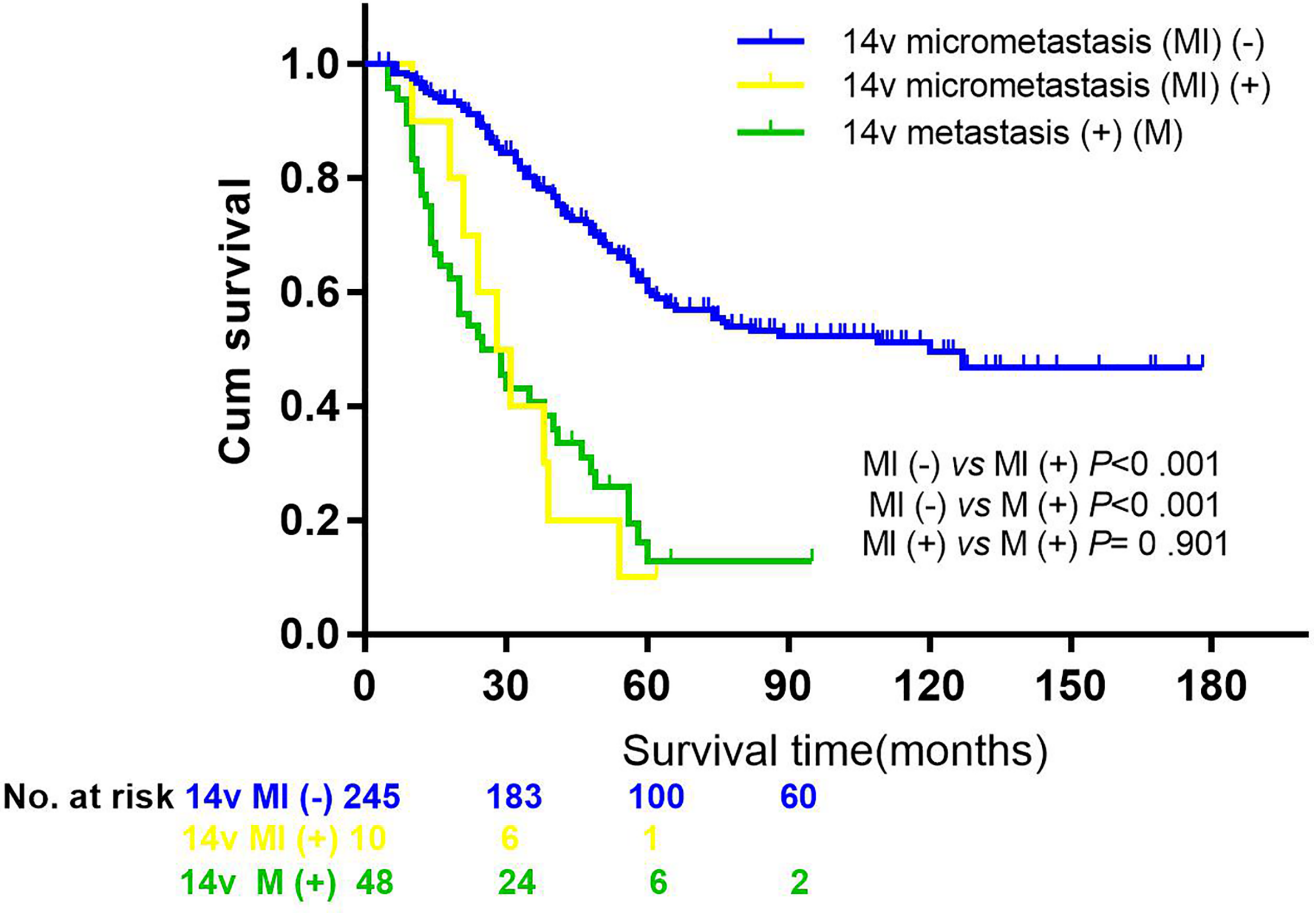
Comparisons of overall survival by 14v metastasis or micrometastasis status in patients with GC. Kaplan-Meier curves were employed to analyze the difference between groups (the group without 14v micrometastasis or metastasis *vs* the group with 14v micrometastasis, *P* < 0.001, HR = 2.093, 95% CI = 1.480–2.961; the group without 14v micrometastasis or metastasis *vs* the group with 14v metastasis, *P* < 0.001, HR = 3.931, 95% CI = 2.671–5.787; the group with 14v micrometastasis *vs* the group with 14v micrometastasis, *P* = 0.901, HR = 1.047, 95% CI = 0.501–2.171).

**Figure 3:**
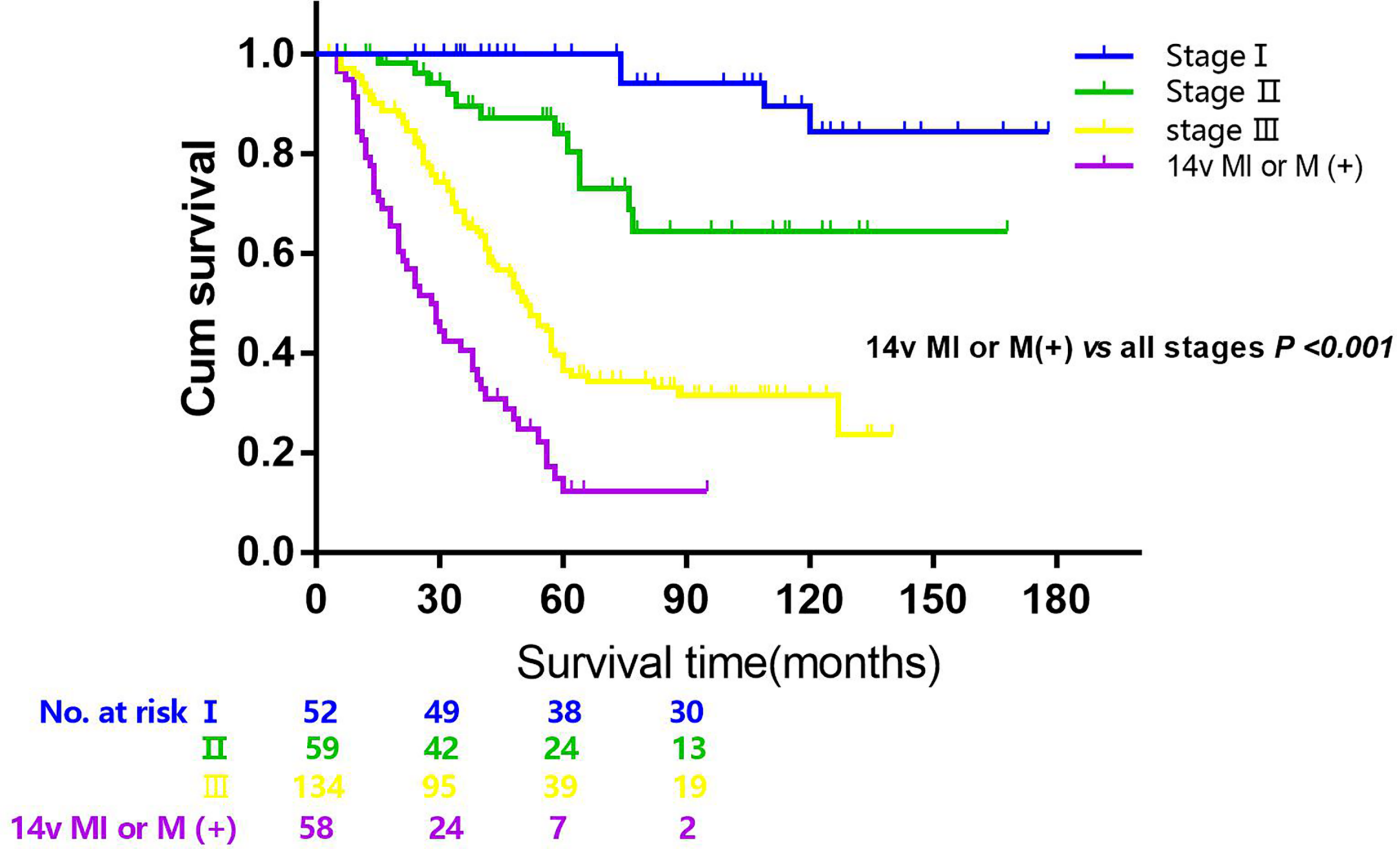
Overall survival after R0 resection categorized by tumor stage and 14v metastatic status (14v (+), 14v metastasis or micrometastasis; stage I, II, III). *P < *0.001 (the group of 14v (+) *vs* the group of all stages, 14v (−), log-rank test).

The 5-year overall survival rate of patients with LN14v metastasis and LN14v micrometastasis was 12.9 and 10.0%. The 5-year survival rate of patients in the positive group (LN14v micrometastasis or metastasis) was 12.4%. The negative group (with neither LN14v metastasis nor micrometastasis) had a more favorable survival in comparison to the positive group (*P* = 0.000, HR = 4.001, 95% CI, 2.789–5.739, **Figure 1**). In stratified analysis, the negative group had a higher 5-years overall survival rate (60.1%) than that those in the group with LN14v micrometastasis or metastasis (*P* < 0.001, HR=2.093, 95% CI,1.480–2.961; *P* < 0.001, HR=3.931, 95% CI, 2.671–5.787, **Figure 2**). The difference between patients with LN14v micrometastasis and LN14v metastasis was not significant (*P*=0.901, HR = 1.047, 95% CI, 0.501–2.171). Univariate analysis results showed age, gender, tumor size, Borrmann type, pT stage, pN stage, pTNM stage, and pathological type were correlated with the prognosis. Furthermore, multivariable Cox proportional hazard model analysis demonstrated that LN14v metastatic status (*P* = 0.001, HR = 1.936, 95% CI, 1.323–2.834), pT stage (*P* = 0.003, HR = 2.725, 95% CI, 1.416–5.244), pN stage (*P* < 0.001, HR=2.090, 95% CI, 1.688–2.588), pathological type (*P* = 0.043, HR = 1.448, 95% CI, 1.012–2.072), and Bormann type (*P* < 0.001, HR = 1.341, 95% CI, 1.148–1.566) were significant prognostic variables (**Table 6**). Notably, patients who underwent radical gastrectomy plus the LN14v dissection, and those with LN14v metastasis or micrometastasis, had worse survival than patients in stages I, II, and III with neither LN14v metastasis nor micrometastasis (*P* < 0.001, **Figure 3**).

**Table 6:** Univariate and multivariate analysis of overall survival in patients with gastric cancer undergoing radical gastrectomy plus 14v dissection.

Variables	Univariate analysis			Multivariate analysis		
	*β*	HR (95% CI)	*P* value	*β*	HR (95% CI)	*P* value
Gender	0.354	0.702 (0.503–0.980)	0.037	0.081	0.922 (0.773–1.110)	0.366
Age	0.378	1.459 (1.052–2.023)	0.024	0.268	1.307 (0.932–1.832)	0.12
Tumor size	0.919	2.506 (1.761–3.567)	0.001	0.04	1.040 (0.695–1.557)	0.847
Location	0.09	1.094 (0.823–1.454)	0.537			
Borrmann type	0.409	1.505 (1.294–1.751)	0.001	0.293	1.341 (1.148–1.566)	0.000
Postoperative T category (pT)	1.757	5.797 (3.267–10.286)	0.001	1.003	2.725 (1.416–5.244)	0.003
Postoperative N category (pN)	0.908	2.480 (2.058–2.989)	0.001	0.737	2.090 (1.688–2.588)	0.000
Micro- or metastasis Status of 14v	1.386	4.001 (2.7894–5.739)	0.000	0.661	1.936 (1.323–2.834)	0.001
Histological type	0.582	1.789 (1.269–2.524)	0.001	0.37	1.448 (1.012–2.072)	0.043
Grade of differentiation	0.542	1.719 (1.101–2.684)	0.017	0.102	1.107 (0.679–1.806)	0.684
Extent of resection	0.461	1.586 (1.107–2.271)	0.012	0.264	1.302 (0.883–1.921)	0.183

### The Benefit of Lymphadenectomy of LN14v in Gastric Cancer

**Figure 4:**
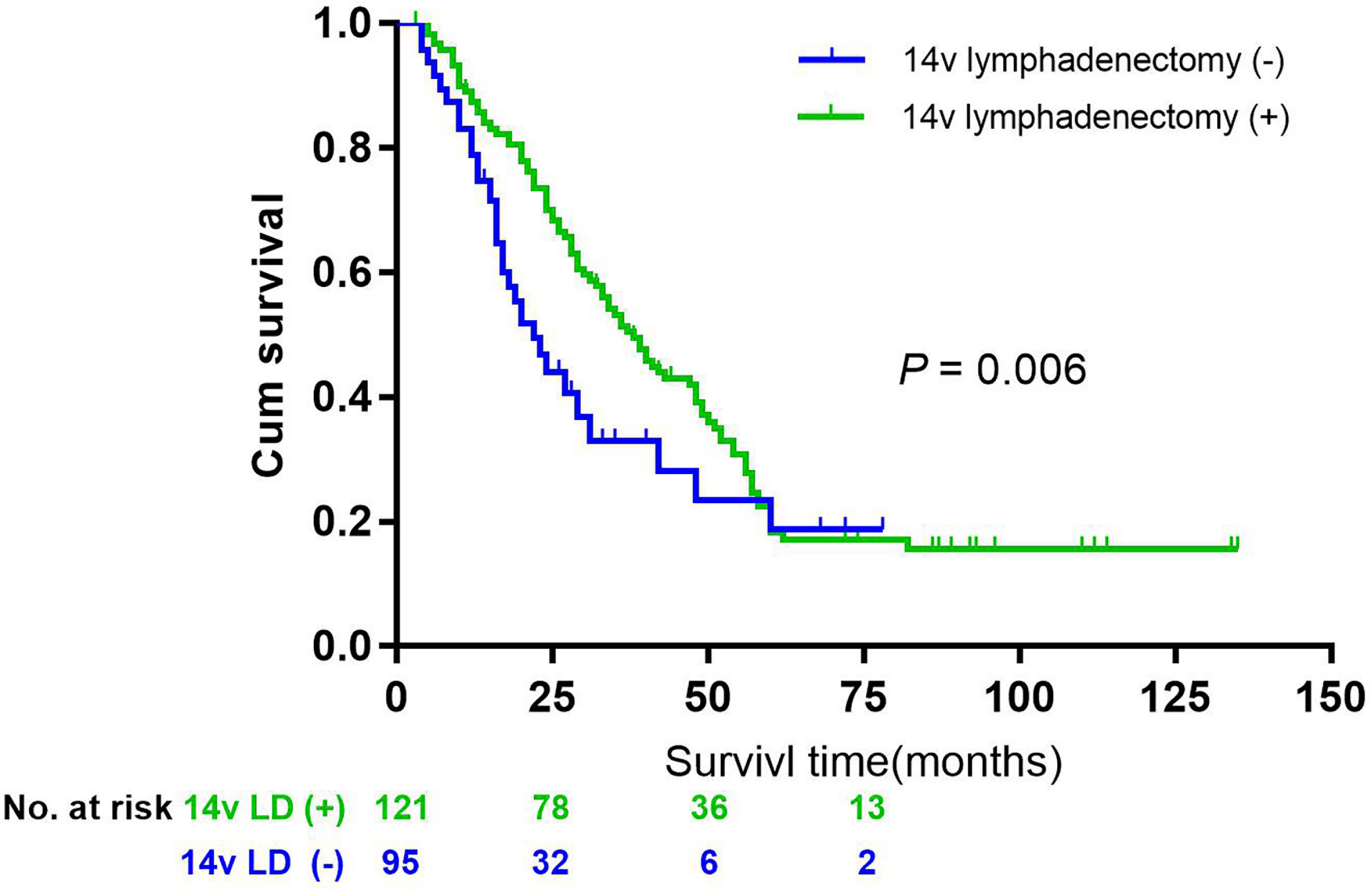
Overall survival after R0 resection categorized by lymphadenectomy of 14v in patients with gastric cancer of stage III, L or Middle area, pN2-3, and LN 6(+), *P* = 0.006.

Having established that 14v metastatic status was of prognosis significance for adequately staged patients treated by radical gastrectomy plus the 14v dissection, we sought to identify patient subgroups for whom the benefit was maximized and those for whom was it was not of prognostic significance. Assuming that the metastasis or micrometastasis of 14v was independently associated with site, Borrmann classification, postoperative lymph node metastasis (pN), the metastasis of LN6, the study made comparisons of outcomes between different groups. In matched analysis, patients with gastric cancer of stage III, L/M area, pN2-3 and LN 6(+), underwent lymphadenectomy of 14v had better survival than those without lymphadenectomy of 14v (*P* = 0.006, **Figure 4**).

## Discussion

Immunohistochemical staining for cytokeratin nodes CK8/18 to evaluate micrometastases has been reported in other fields (16). This study used CK8/18 to evaluate micrometastases in lymph nodes to identify clinicopathological characteristics and prognosis of GC with metastasis or micrometastasis to LN14v, and it demonstrated location, pN stage, Bormann type, and the LN6 metastatic status were predictive factors for LN14v metastasis or micrometastasis, implying that tumors located in the M or L area, with stage pN3a or N3b, Bormann III or IV subtypes, and metastasis in LN 6 were likely to be presented with LN14v metastasis or micrometastasis. The results identified a patient subgroup who may obtain maximum benefit from LN14v dissection and those for whom LN14v dissection seemed not to be of prognostic significance. Second, it revealed that the micrometastatic status of LN14v lymph modes is one of the important prognostic factors. Lymph node micrometastasis could provide more accurate prognostic information for patients with GC. Thus, immunohistochemical detection of micrometastasis of lymph nodes is recommended.

Whether LN14v metastasis was associated with regional lymph node (local disease) or systemic disease is a contentious issue (17, 18). When compared to those with locoregional lymph node metastasis, patients with LN14v metastasis had the worst 5-year survival rate (<10%), and it was similar to that of LN16 metastasis, which was categorized as stage IV, implying its systemic disease role. However, several studies demonstrated some patients with LN14v metastasis benefited from LN14v dissection, which prolonged their survival—indicating at least some patients with LN14v metastasis had local rather than systemic disease (17). According to the 2^nd^ edition of the Japanese Classification of Gastric Carcinoma, LN14v dissection was included in the N2 group for tumors located at the lower third of the stomach (19). However, it was once classified as M1 status in the 3^rd^ edition of the Japanese Classification of Gastric Carcinoma, which recommended it was unnecessary to dissect LN14v for patients with GC. Furthermore, the 3^rd^ edition of the Japanese GC treatment guidelines 2010 proposed patients with tumors located in the lower third of the stomach with LN6 metastasis need dissection of LN14v (20). Therefore, during evaluations of whether it is essential for patients with GC to undergo dissection of LN14v, the LN14v metastatic rate, the clinicopathological features associated with LN14v metastasis, the security and feasibility of LN14v dissection, and the significance of dissection should be considered.

Lymphatic metastasis is considered to spread *via* lymphatic flow from the primary tumor site, and the lymphatic flows from any particular point have some preferred pathway (21–24). There are three lymphatic pathways in the region of the lower stomach. The lymphatic drainage from LN6 directly flows to LN14v, and then lymphatic flow reaches LN16, which finally joins the thoracic duct. In terms of lymphatic flow pathways, LN6 is anatomically upstream of LN14v, whose metastatic status is very closely correlated to that of LN6. A previous study investigated the impact of regional nodes’ metastatic status on LN14v metastasis (17). It revealed that the metastatic status of LN6 were predictive factors for LN14v metastasis. Our study was consistent with the previous research and the stepwise lymphatic metastasis theory. In our study, we found LN6 metastatic status was a significant independent variable for the metastatic status of LN14v. Of 146 patients with LN6 metastasis, 34.2% cases presented with the metastasis or micrometastasis in LN14v. Similar to previous studies, the LN6 status predicted the absence of LN14v metastases, with a low false-negative rate (10.7%). In addition, the study also demonstrated tumor site, Borrmann classification, and pN stage were also correlated with LN14v metastasis or micrometastasis. Tumors located in the region of the low or middle stomach and presented with Borrmann III/IV subtype and stage pN2-3 seem likely to metastasize or micrometastasize to LN14v.

Interactions among various factors promote the occurrence and development of GC, which has complicated biological characteristics, high heterogeneity, and poor prognosis (25). One study reported that the 5-year survival rate of patients with GC with lymph node metastasis to LN14v was extremely low (11.3%), which was also described in another study with a poor 5-year survival rate (9.0%) (26, 27). Our study provided a comparable result in that patients with LN14v metastasis or micrometastasis had unfavorable prognosis, and the 5-year survival rate was 12.4%. It should be noted that patients with LN14v metastasis or micrometastasis had a lower 5-year survival rate in comparison with those who had regional lymph node metastasis. However, according to Sasako’s therapeutic index theory, the therapeutic index of LN14v dissection is 2.1 in lower-third GC—which was comparable to that of LN12a dissection (2.7), the N2 group lymph node dissection (28). Although the benefits to patients from LN14v dissection vary, it is important to distinguish those who would benefit from LN14v dissection. Eom et al. noted that even if patients with LN14v metastasis had an unfavorable prognosis, LN14v dissection could improve overall prognosis, especially in those with tumor sites located in the middle or lower area of the stomach, positive LN6 lymph nodes, and clinical stage III/IV gastric cancer (17). Our study revealed tumor site, Borrmann classification, and pN stage were significantly correlated with LN14v metastasis or micrometastasis, which was consistent with the previous study. In a matching analysis, we also suggested patients with stage III GC, L/M location, stage pN2-3, and LN6(+) who underwent lymphadenectomy of LN14v had better survival than those without lymphadenectomy of LN14v.

At present, detection methods for lymph node micrometastasis mainly include serial sections, PCR, and immunohistochemistry. Although serial sections can significantly improve the detection of lymph node micrometastasis, it is difficult to promote it in clinical practice because the procedure is difficult and time-consuming. PCR is characterized by high sensitivity and specificity in detecting lymph node micrometastasis, but the requirement for fresh samples, the relatively complicated operation process, and high costs hinder its routine application in clinical pathological diagnosis. Compared with previous ones, the immunohistochemical method seems to be more useful in clinical work. Cytokeratin is one component of the cytoskeleton of epithelial cells and is not present in normal lymph nodes. Ishii et al. reported that using CK8/18 monoclonal antibody is one accurate method to detect lymph node micrometastasis in gastric cancer (29). In 35 patients in whom micrometastasis was detected in the lymph nodes, the positivity rate for CK8/18 monoclonal antibody testing was 11.4%. Our study suggests that the micrometastasis rate in LN14v is only 3.9%. The discrepancy may result from different intervals between serial sections, the harvested number of sections from different paraffin-embedded blocks, the included cases, and the focused lymph node.

Although the majority of studies have demonstrated that patients with lymph node micrometastasis have a worse prognosis, whether lymph node micrometastasis results in postoperative recurrence or metastasis and consequently affects patients’ prognosis remains controversial (30–32). Micrometastases can be promoted or inhibited by various factors, such as the host’s immune status, postoperative radiotherapy or chemotherapy, and tumor microenvironment. In accord with a previous study, we showed that micrometastatic status is a significant variable associated with patient survival. However, the difference between survival of patients with LN14v micrometastasis and that of LN14v metastasis was not obvious, which may result from the limited samples of positive micrometastatic cases. Additional multicenter, randomized trials are required to further investigate the extent of the impact of micrometastatic status on survival in GC. This study shows that micrometastatic status can be considered as one promising prognostic predictor in GC that can provide accurate pathological staging and treatment guidelines. We recommend patients with LN14v micrometastasis, or who are suspected of having LN14v micrometastasis, undergo LN14v dissection.

Our study has some limitations, mainly because it is a retrospective case-control study with a limited number of participants. Therefore, first, the small sample size may produce selection bias. Second, in comparison with the previous study, different interval between serial sections and the harvested number of sections from different paraffin-embedded blocks might have an effect on the apparent micrometastatic rate in LN14v. Third, although the study revealed that LN14v metastatic or micrometastatic status was an independent risk factor associated with survival, it was not a randomized study and could not clarify the impact of LN14v dissection on survival for patients with LN14v metastasis or micrometastasis.

In conclusion, locally advanced gastric carcinoma, located in the middle or lower stomach area with LN6 metastasis, is likely to have metastasis or micrometastasis in LN14v, and lymphadenectomy of LN14v might improve the survival of patients with stage III GC, located in the lower or middle area, stage pN2-3, and LN 6(+) if serious complications of LN14v dissection can be sufficiently controlled. Micrometastatic status of LN14v can be considered as one promising prognostic predictor for GC.

## Data Availability Statement

The raw data supporting the conclusions of this article will be made available by the authors, without undue reservation.

## Ethics Statement

The studies involving human participants were reviewed and approved by the Ethics Committee of Liaoning Cancer Hospital. The patients/participants provided their written informed consent to participate in this study.

## Author Contributions

XX searched and reviewed the literature, designed the research, and wrote the paper. GZ designed the research. YZ performed statistical analysis. TZ reviewed the literature and interpreted the paper. ZZ participated in reviewing the literature, designing the research, revising and writing the paper. All authors contributed to the article and approved the submitted version.

## Conflict of Interest

The authors declare that the research was conducted in the absence of any commercial or financial relationships that could be construed as a potential conflict of interest.

## Publisher’s Note

All claims expressed in this article are solely those of the authors and do not necessarily represent those of their affiliated organizations, or those of the publisher, the editors and the reviewers. Any product that may be evaluated in this article, or claim that may be made by its manufacturer, is not guaranteed or endorsed by the publisher.
