# Supplementary material for: Clinical Significance of Metastasis or Micrometastasis to the Lymph Node Along the Superior Mesenteric Vein in Gastric Carcinoma: A Retrospective Analysis
Source: Front Oncol. 2021 Jul 29;11:707249. doi: 10.3389/fonc.2021.707249 (PMC8358673; doi:10.3389/fonc.2021.707249)
Supplement: Supplementary file 1 [file DataSheet_1.doc]

Supplementary materials

Figure legends

Figure 1. Flow chart illustrating the study selection process.

Figure 2. a. The positive control group (micrometastasis detection of the tumor tissues of GC lesion by immunohistochemical staining for cytokeratin CK8/18; field of vision, ×100 times). b and c, the negative cases of 14v micrometastasis (immunohistochemical staining for cytokeratin CK8/18; field of vision, ×100 times; at the top right site, ×200 times).

Figure 1.


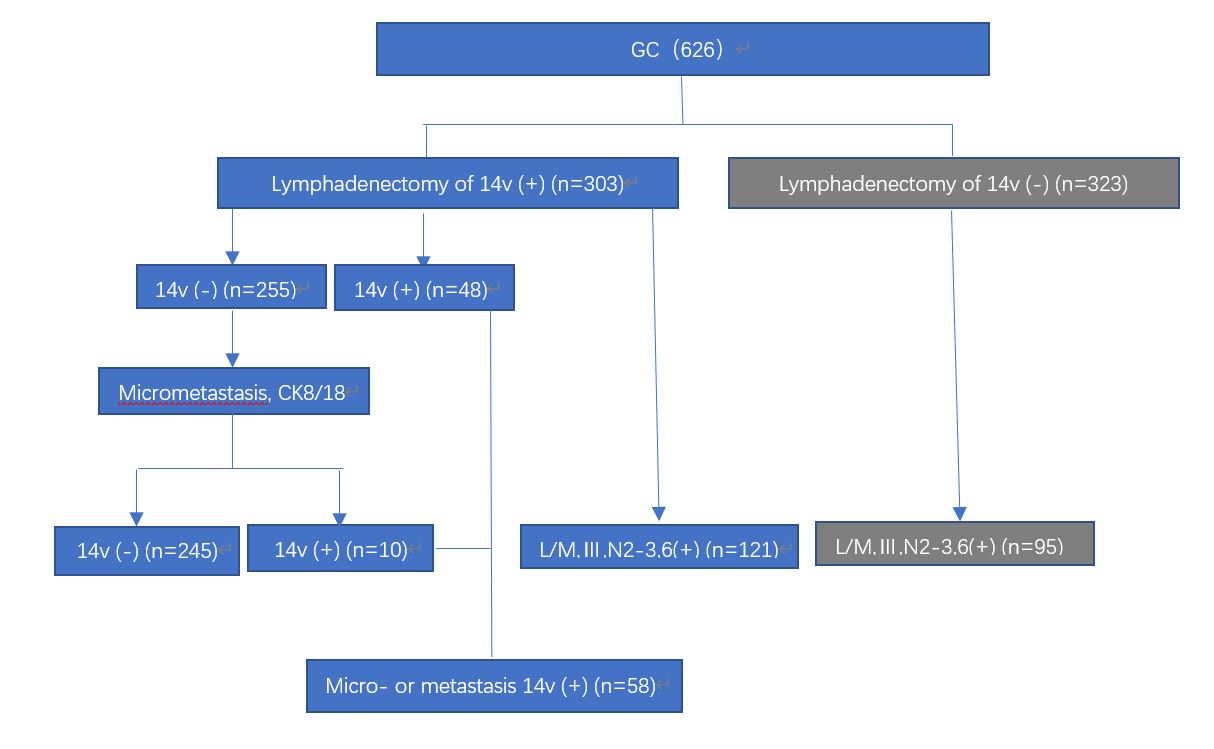


Figure 2.

a


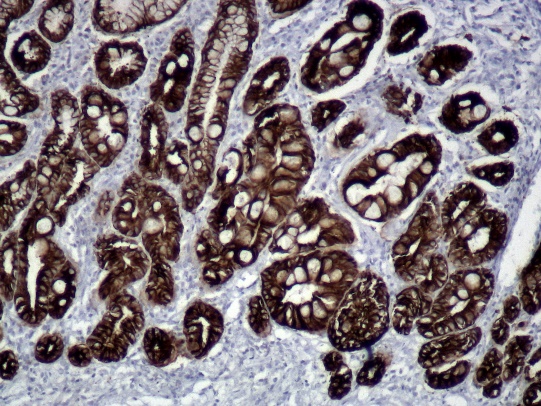


b


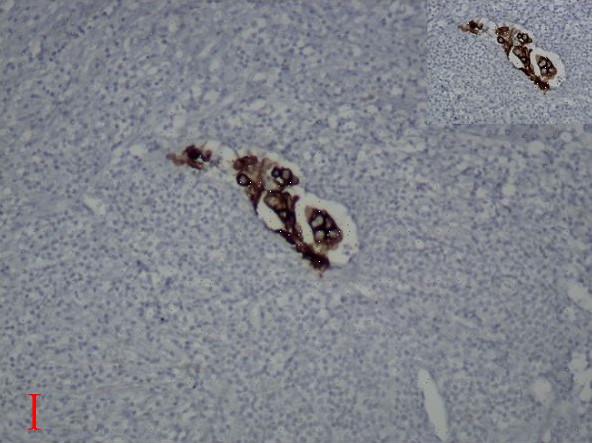


c


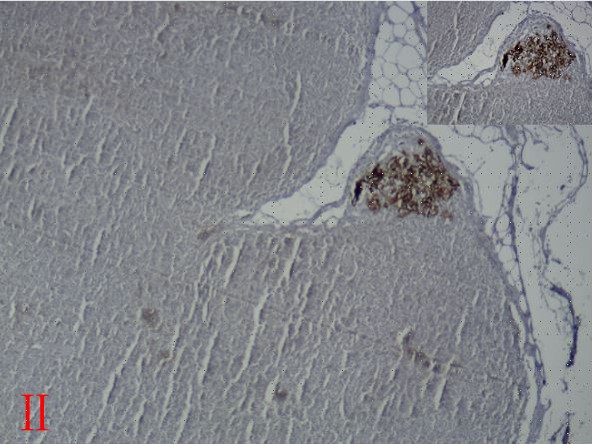
l
